# Outcomes of the Posterior Approach for the Treatment of Radial Head Fractures and Associated Elbow Injuries: A Retrospective Observational Study

**DOI:** 10.7759/cureus.34041

**Published:** 2023-01-21

**Authors:** Shakti Prasad Das, Govind VJ, Preethiv R, Suhas Sondur, Avinash Naik, Ankit Gulia, Anwesit Mohanty

**Affiliations:** 1 Orthopedics, Kalinga Institute of Medical Sciences, Bhubaneswar, IND

**Keywords:** coronoid fracture, radial head injuries, post-traumatic stiff elbow, a posterior approach, terrible triad injuries, radial head fracture

## Abstract

Background: The lateral approach to the radial head remains the routinely used approach for surgical fixation or replacement despite the risk of injury to lateral soft tissue structures. Multiple approaches are required when dealing with complex elbow injuries involving other bony and soft tissue structures which lead to greater soft tissue dissection, prolonged immobilization, and a higher rate of elbow stiffness. This article utilizes a single posterior approach involving the Boyd interval in the surgical management of radial head fractures with an associated elbow injury.

Methods: Thirteen patients with radial head fractures and related elbow injuries treated with the posterior approach to the elbow were retrospectively analyzed. All patients were operated on by a single surgeon and followed up for a minimum of 18 months postoperatively. Functional evaluation of the patients was performed at the final follow-up which comprised a range of movements of the elbow, visual analogue scale (VAS), Disability of Arm, Shoulder, and Hand (QuickDASH), and the Mayo Elbow Performance Score (MEPS).

Results: The mean VAS score was 2.16, QuickDASH score, and Mayo elbow score were 7.15 ± 2.96 and 78.46 ± 8.26 respectively. The flexion-extension arc of the elbow was 128.46 ± 4.27 degrees and the supination-pronation arc was 133.92 ± 4.04 degrees at one-year follow-up. Two patients developed early postoperative complications (elbow stiffness and ulnar nerve neuropraxia) and recovered spontaneously. No patients developed neuropraxia of the posterior interosseous nerve (PIN).

Conclusion: The single incision posterior (Boyd) approach to the elbow offers complete access to the radial head, olecranon, coronoid, and lateral ligamentous structures in complex elbow injuries and provides good functional outcomes in our small observational study.

## Introduction

Radial head fracture is one of the most common intra-articular fractures of acute elbow injuries accounting for 1-4% of all fractures in adults and 20-30% of all elbow fractures [1]. It could be associated with an episode of elbow instability, a mechanical obstruction of elbow motion, a dislocation of the distal radioulnar joint, or even an injury to the interosseous membrane (Essex-Lopresti injury) [2]. Fractures of the capitulum, olecranon, and coronoid with or without elbow dislocation (terrible triad injuries) are commonly associated with radial head fractures [3]. The optimum surgical strategy for treating a radial head fracture at the elbow has been reported using a variety of surgical techniques, and this topic is never settled. The posterolateral approach (Kocher) and the lateral approach (Kaplan) are a few examples. The Kocher approach involves an interval between the anconeus and extensor carpi ulnaris (ECU) whereas the Kaplan interval uses the interval between extensor carpi radials brevis (ECRB) and extensor digitorum communis (EDC) [4]. During surgery on the radial head, there is always a chance of damaging the lateral elbow structures such as the lateral ulnar collateral ligament (LUCL), common extensor origin, or the posterior interosseous nerve (PIN) even while performed by experienced surgeons [5]. Despite the well-known complications, the lateral approach remains the fairly commonly used approach for the radial head.

A problem arises when a radial head fracture necessitating surgery is associated with an olecranon fracture, coronoid fracture (in terrible triad injuries), or a medial ligamentous injury. A separate incision is required to approach the associated injury which can lead to various local surgical complications. Prolonged immobilization, delayed wound healing, greater soft tissue damage, and resultant elbow stiffness are associated with multiple incisions to approach elbow injuries [6]. A single posterior approach can be used as universal access to the radial head, proximal ulna, and distal humerus along with the capsule-ligamentous structures. An olecranon osteotomy may be performed to visualize the elbow joint but when the injury is associated with an olecranon fracture, access to the radial head can be further eased through the fracture line as an interval [5].

Hence, we are utilizing the posterior approach (Boyd) in which the radial head, olecranon fracture, or coronoid fracture can be addressed. This approach involves a lesser chance of PIN injury, and early wound healing, and also has the advantage of extending the incision more distally than the lateral approach.

## Materials and methods

Thirteen individuals with radial head fractures and related elbow injuries operated on between June 2018 and July 2021 were retrospectively evaluated. All patients were operated on by a single orthopedic surgeon using the posterior approach of Boyd and were included in the study. All cases underwent surgery within a week of the injury. Patients treated with additional approaches to the elbow were excluded from the study.

Operative technique

**Figure 1:**
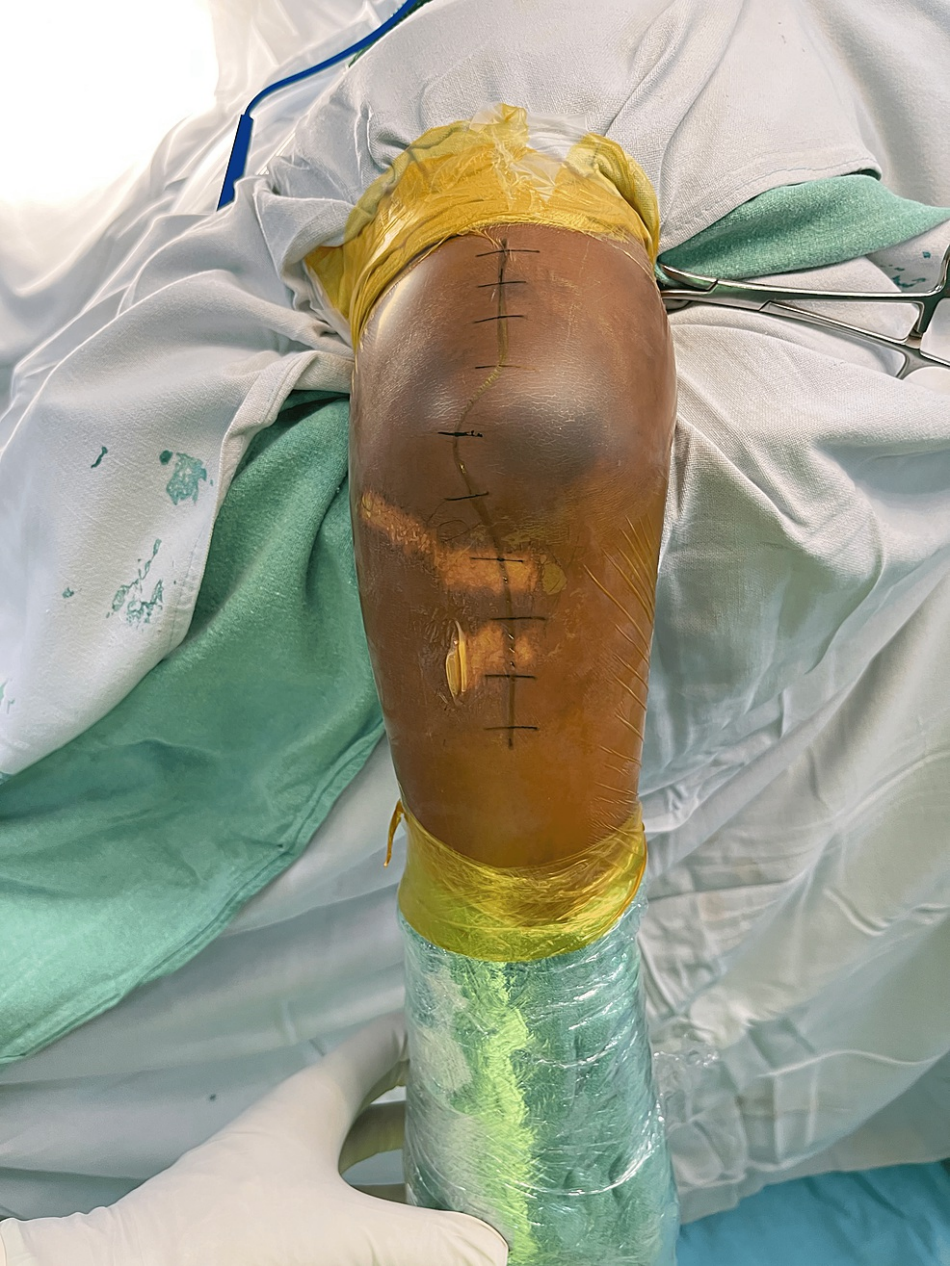
Patient is placed in lateral decubitus with the affected side upwards and limb supported on an arm cushion. Incision placed along the subcutaneous border of ulna and lateral border of triceps gently curved around the olecranon.

All patients were operated on under regional or general anesthesia. The afflicted limb was supported on a cushioned arm support with the patient lying in a lateral decubitus position. A long longitudinal skin incision is made moving from the lateral border of the triceps to the subcutaneous border of the ulna and gently curved laterally around the tip of the olecranon (Figure 1).

**Figure 2:**
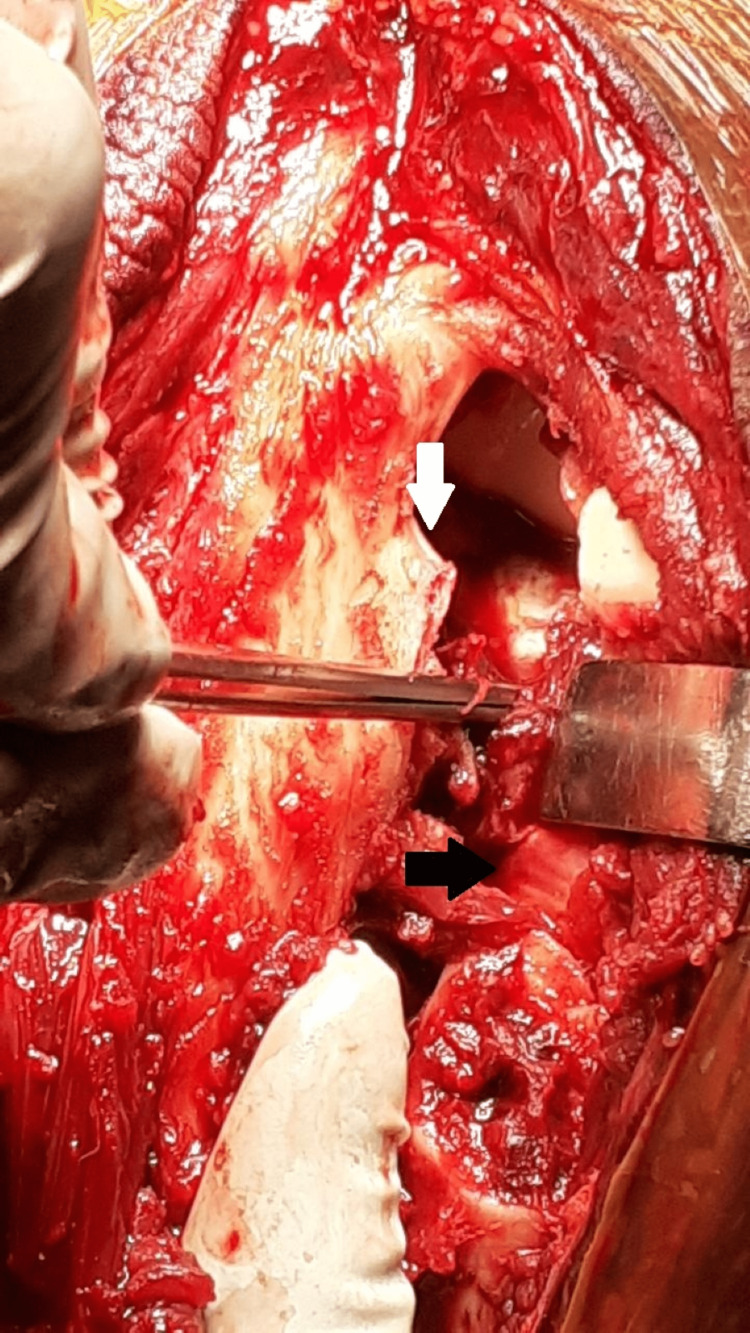
Deep dissection to expose the fascia between the anconeus and ECU. Fascia is incised retaining a small cuff of the ulnar attachment intact (white arrow). Deep into this layer, the LUCL, annular ligament, and posterior capsule were found torn (black arrow). The radial head and coronoid are visible through this rent. ECU - extensor carpi ulnaris, LUCL - lateral ulnar collateral ligament

Full-thickness flaps are elevated from the deep fascia. Along with the skin incision, the deep posterior fascia of the arm and forearm is incised longitudinally. The posterior cutaneous nerve of the forearm lies just lateral to the skin incision at this level and must be preserved during the deep dissection. The ulnar nerve is routinely not exposed as we approach the radial head lateral to the border of the triceps, but it is exposed and isolated initially before the deep dissection in cases of olecranon or distal humeral fractures. The fascia between the anconeus and the ECU is incised longitudinally and the small width of the ulnar attachment of the fascia is retained. To visualize the supinator lying deep, the anconeus and ECU are elevated subperiosteally. The supinator can also be raised sub-periosteally from the ulna. The LUCL, the joint capsule complex, and the annular ligament are located deep within this layer (Figure 2).

**Figure 3:**
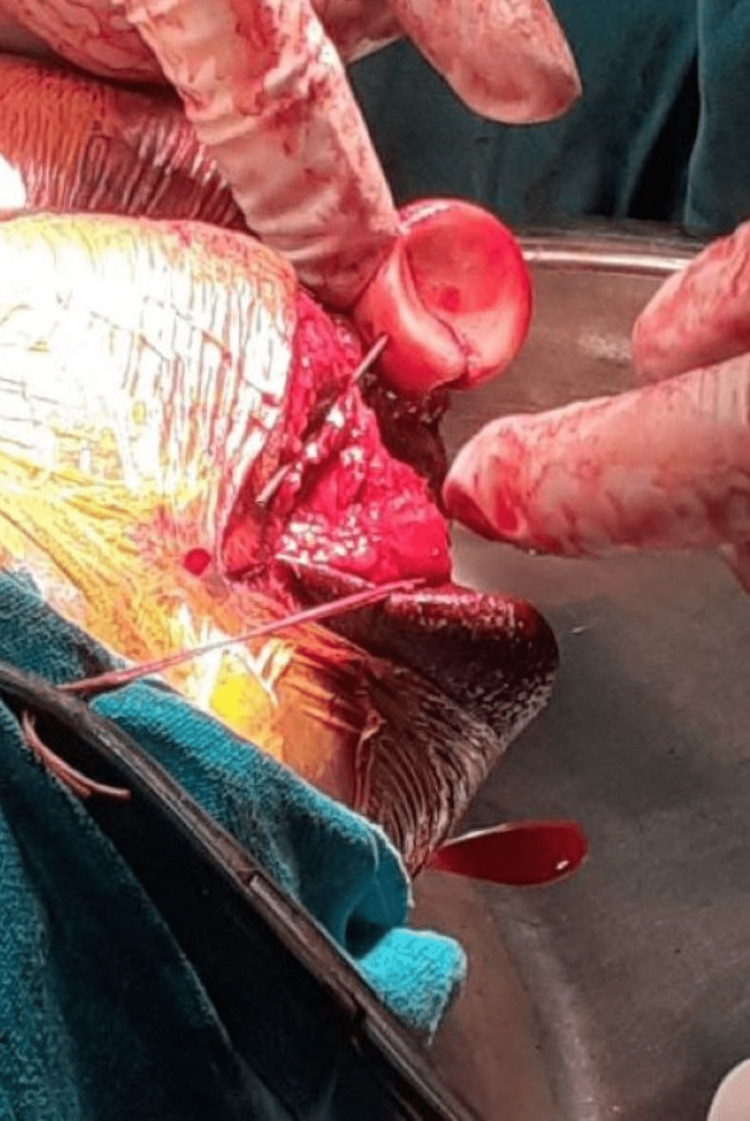
The radial head is delivered posteriorly and internal fixation is done with headless compression screws.

Deep to these, it is simple to palpate the radial head. These ligaments along with the posterior capsule may be torn in posterior dislocations of the radial head or terrible triad injuries making exposure of the radial head relatively easier. Sharp dissection is used to remove the ligaments and capsule directly from the ulna at the supinator crest and deliver the radial head posteriorly. The PIN is particularly at risk of injury in the lateral and posterolateral approaches due to prolonged retraction. This complication is prevented by performing the posterior approach. The radial head is fixed either by anatomical plate or using Herbert screws in a tripod manner or a radial head replacement (RHR) is performed in severely comminuted fractures (Figure 3).

**Figure 4:**
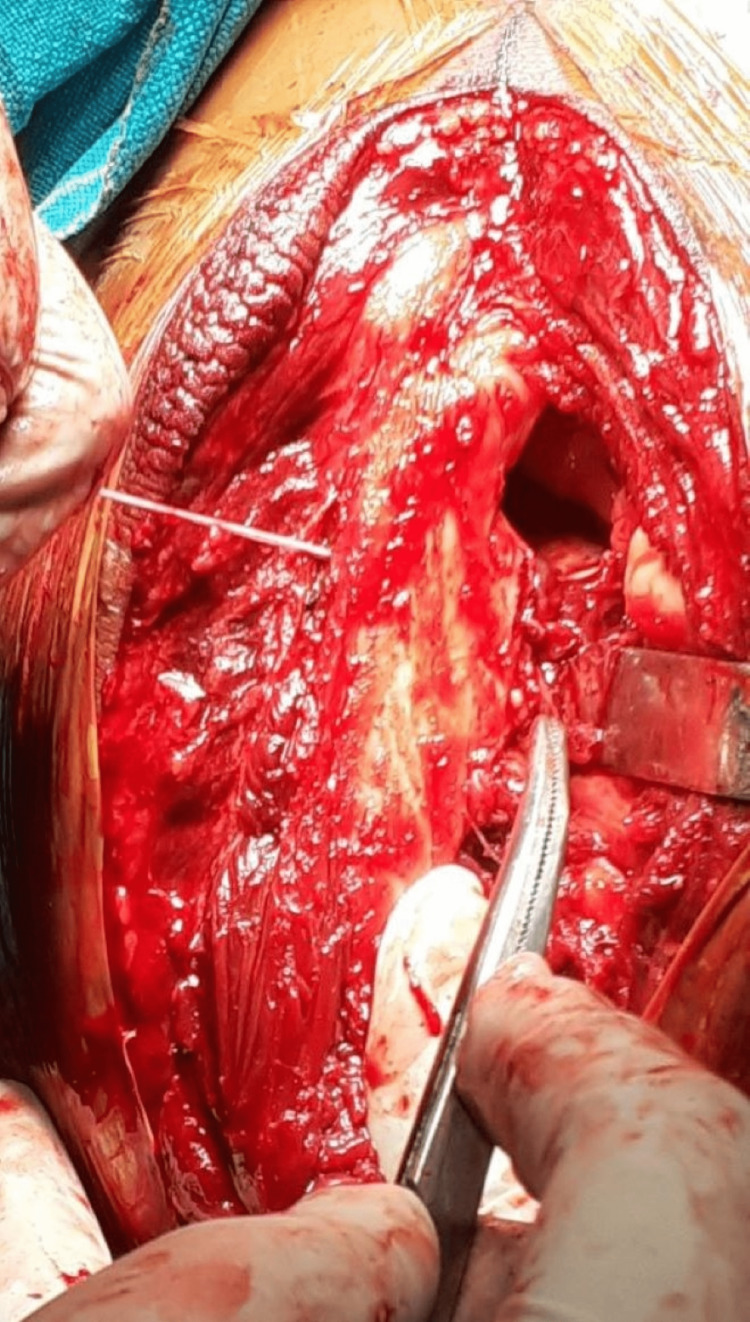
The anterior capsule and coronoid are repaired by taking bites with a FibreWire suture and passing it through the bony tunnel made in the olecranon.

In terrible triad injuries, access to the anterior capsule and coronoid can be created by dislocating the elbow joint posteriorly. Sharp dissection is used to reveal the lateral tissues, and the elbow is dislocated by avulsing the common extensor origin from its origin. The anterior capsule and coronoid process are exposed and the coronoid fragment is fixed using FiberWire sutures (Arthrex Inc., Florida, USA) (Figure 4).

**Figure 5:**
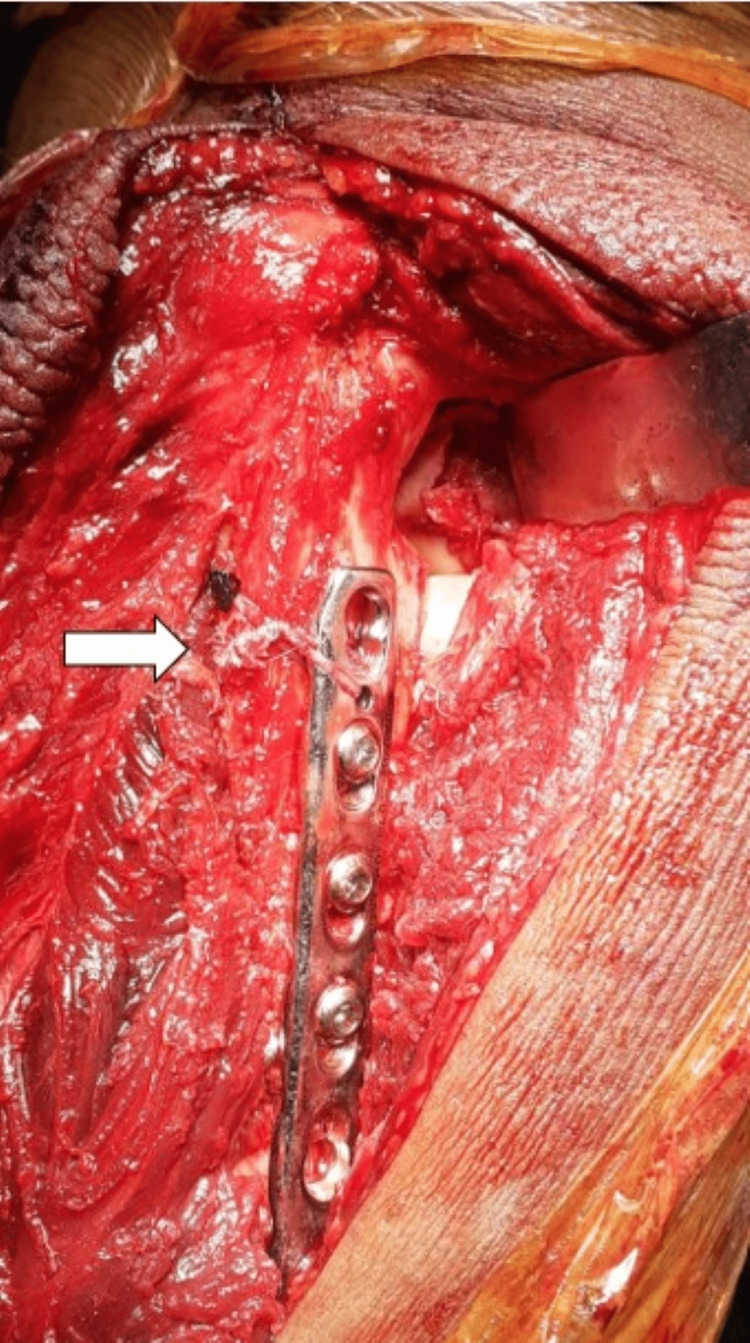
The proximal ulna fracture is fixed with an LCP and coronoid sutures (white arrow) tied over the dorsal aspect of the olecranon and the plate. LCP - locking compression plate

The sutures are taken through the capsule and shuttled through a bony tunnel created through the coronoid base to exit dorsally and held in place by knots (Figure 5).

This repair is done at 30° of elbow flexion. The LUCL reconstruction is done using anchor sutures placed into the supinator crest of the ulna. Bony tunnels are created through the ulna in the correct anatomic position of the ligamentous structures to provide optimum elbow stability. The avulsed lateral structures are then repaired back to the lateral epicondyle using a suture anchor. The fascia is closed with the number 1 vicryl suture (Ethicon, J&J, New Jersey, USA).

The elbow is put through various degrees of flexion and extension and forearm rotation to confirm stability clinically and radiographically.

Rehabilitation

All patients are immobilized in an arm sling for six weeks. Active elbow pronation, supination, flexion, and extension were started immediately from postoperative day one. The patients were reviewed at two weeks, evaluated clinically and radiologically, and sutures were removed. Physiotherapy was advised as necessary. Flexion and extension are restricted till 30° short of full extension. Sling was discontinued at six weeks and regular activities resumed.

Evaluation of outcomes

All patients were followed up for a minimum of 18 months postoperatively. Functional assessment was performed by two independent authors not involved in the initial surgical management. The assessment consisted of the visual analogue scale (VAS), the Quick Disability of Arm, Shoulder, and Hand score (QuickDASH), and the Mayo elbow performance score (MEPS) [7,8]. The range of movement was assessed by clinical evaluation of the elbow flexion and extension arc and the pronation and supination arc using a goniometer. The QuickDASH score ranged from 0 (no disability) to 100 (full disability) and the MEPS was calculated out of 100 with a score above 90 being excellent, 75-89 good, 60-74 fair, and below 60 as poor outcomes.

## Results

The senior author, a consultant orthopedic surgeon carried out all of the operations. The patient details are mentioned in the following table (Table 1).

**Table 1:** Patient details and functional outcomes. DOS - date of surgery, RH - radial head, ORIF - open reduction and internal fixation, RHR - radial head replacement (uncemented), TBW - tension band wiring, LUCL - lateral ulnar collateral ligament, QuickDASH - Disability of Arm, Shoulder, and Hand, MEPS - Mayo elbow performance score * - LCP fixation ^ - Precontoured olecranon plate fixation

No. of operations	Age	DOS	Diagnosis	Treatment	VAS score (after one-year follow-up)	Flexion-extension arc	Supination and pronation	QuickDASH score	MAYO score	Complications
1	19	Jun-18	Right radial head fracture with olecranon fracture	ORIF RH + Olecranon TBW	nil	0 to 130 deg	0 to 75 deg/0 to 70 deg	4	85	nil
2	43	Oct-18	Right radial head fracture with olecranon fracture	ORIF RH + Olecranon plating*	2	10 to 130 deg	0 to 60 deg/0 to 68 deg	11	65	nil
3	22	Dec-18	Left radial head fracture with olecranon fracture	RHR + Olecranon plating*	3	0 to 125 deg	0 to 62 deg/0 to 70 deg	9	70	stiffness
4	37	Feb-19	Right radial head fracture with olecranon fracture	ORIF RH + olecranon plating^	2	0 to 135 deg	0 to 60 deg/0 to 65 deg	5	80	nil
5	28	Apr-19	Right radial head fracture	ORIF RH	nil	0 to 130 deg	0 to 65 deg/0 to 70 deg	6	75	nil
6	60	Aug-19	Terrible triad of left elbow	RHR + fibrewire suture fixation of coronoid process + LUCL repair with suture anchor	2	0 to 125 deg	0 to 60 deg/0 to 66 deg	12	70	ulnar nerve neuropraxia recovered after three months
7	54	Nov-19	Left radial head fracture	RHR	nil	0 to 130 deg	0 to 65 deg/0 to 70 deg	10	75	nil
8	47	Jan-20	Left radial head fracture with olecranon fracture	ORIF RH + olecranon plating^	nil	0 to 130 deg	0 to 70 deg/0 to 70 deg	7	80	nil
9	29	Mar-20	Terrible triad of left elbow	ORIF RH + fibrewire suture fixation of coronoid process + LUCL repair with suture anchor	nil	0 to 130 deg	0 to 65 deg/0 to 70 deg	5	90	nil
10	42	May-20	Terrible triad of left elbow	ORIF RH + fibrewire suture fixation of coronoid process	2	0 to 135 deg	0 to 60 deg	4	85	nil
11	37	Sep-20	Left radial head fracture with olecranon fracture	ORIF RH + olecranon plating*	nil	0 to 130 deg	0 to 65 deg/0 to 70 deg	3	90	nil
12	61	Feb-21	Left radial head fracture	ORIF RH	nil	5 to 125 deg	0 to 65 deg/0 to 70 deg	7	85	nil
13	52	Jul-21	Right radial head fracture with olecranon fracture	RHR + Olecranon plating^	2	0 to 125 deg	0 to 62 deg/0 to 68 deg	10	70	nil

**Figure 6:**
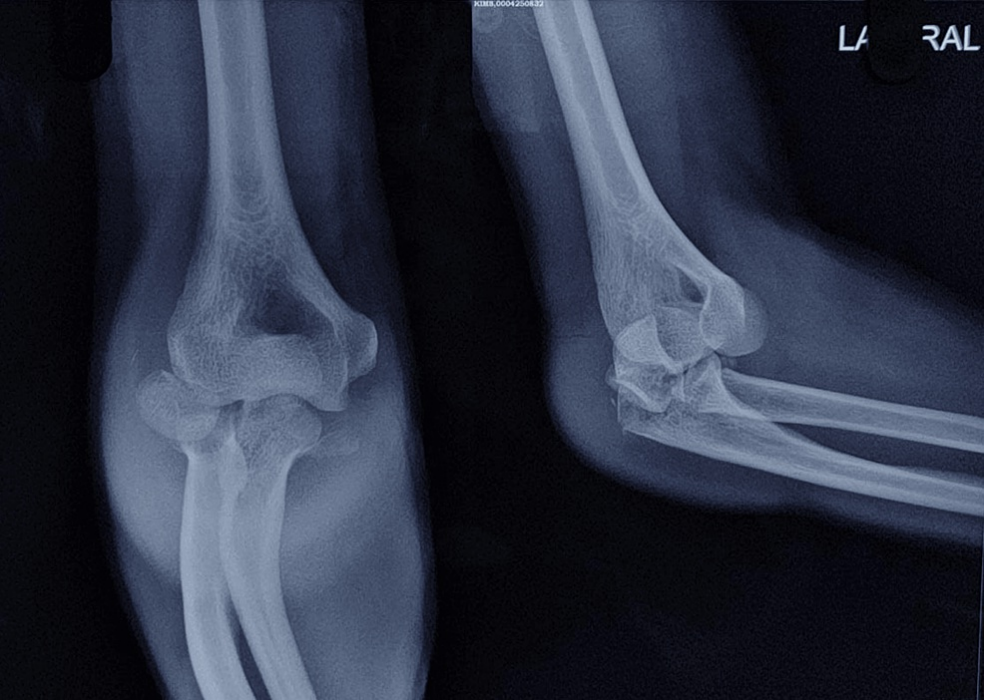
Initial pre-operative radiographs showing a right side radial head fracture associated with a comminuted fracture-dislocation of the olecranon.

**Figure 7:**
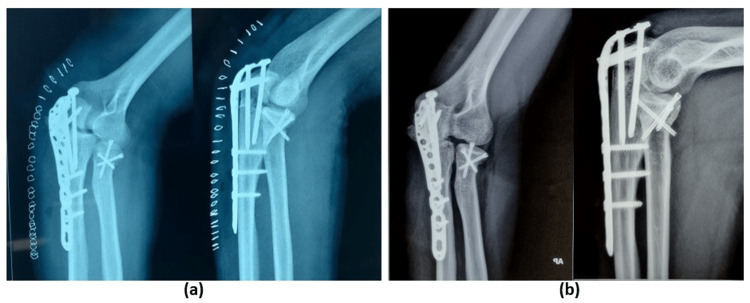
(a) Immediate postoperative radiograph depicting a contoured olecranon plate fixation and radial head fixation with headless cancellous screws (tripod fixation). (b) Twelve-month follow-up with a complete union of fractures.

**Figure 8:**
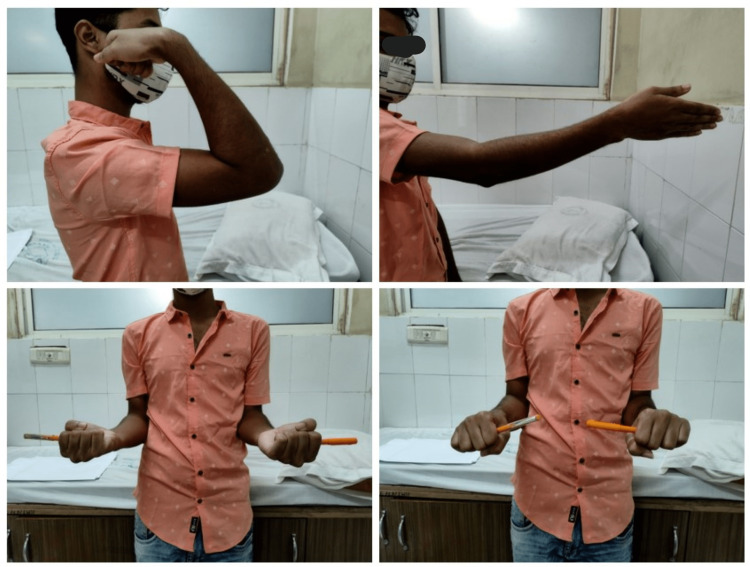
Twelve-month follow-up showing satisfactory flexion-extension and prono-supination.

The average age of the patients was 41. Eight out of the 13 patients were male and five were female. Nine patients underwent internal fixation for the radial head fracture with a mean age of 38 years. The RHR procedure was performed on the remaining four patients, who had a mean age of 47. A case is illustrated below (Figures 6, 7, 8).

All patients were assessed one year following the surgery. The mean VAS score was found to be 2.16. QuickDASH score and MEPS were found to be 7.15 ± 2.96 and 78.46 ± 8.26 respectively. The flexion-extension arc of the elbow was 128.46 ± 4.27 degrees and the supination-pronation arc was found to be 133.92 ± 4.04 degrees at one-year follow-up. All the patients achieved radiological union. One patient suffered from early post-operative stiffness of the elbow which improved with passive physiotherapy and one patient developed an ulnar nerve neuropraxia immediately following surgery which recovered spontaneously at three months. None of the patients in our study suffered from PIN neuropraxia.

## Discussion

Radial head fractures present a relatively common elbow injury pattern for orthopedic surgeons. Various treatment strategies are available based on the severity of the injury. Mason type II fractures are traditionally treated with open reduction and internal fixation whereas, for Mason type III fractures, the optimal treatment still varies. Swensen et al. reviewed outcomes of surgical management of radial head fractures and found an RHR to offer satisfactory results in more comminuted fractures [9].

Several approaches have been described for the radial head, capitellum, proximal radius, or ulna (Table 2).

**Table 2:** Various approaches to the elbow. FCU - flexor carpi ulnaris, PIN - posterior interosseous nerve, LUCL - lateral ulnar collateral ligament, EDC - extensor digitorum communis

Approach	Indication	Advantages	Disadvantages
Kocher’s Approach	Radial Head fractures, Excision, Replacement	Low risk of PIN injury (compared to the Kaplan approach) Easy access to supinator crest of ulna, LUCL May be extended proximally and distally	The distal extension has a risk of injuring PIN
Kaplan Approach	Radial Head fractures, Excision, Replacement	Good view of the anterior half of the radial head LUCL not damaged	High risk of PIN injury
Medial (Over the top) Hotchkiss Approach	Anterior elbow joint exposure, Coronoid fractures	Good view of the coronoid process	Ulnar exploration required Risk of damage to the cutaneous nerve of the forearm
Medial Flexor carpi ulnaris (FCU) split approach	Coronoid fractures	Good view of the coronoid process	Ulnar exploration required Risk of damage to the cutaneous nerve of the forearm
Anterior approach	Anterior neurovascular bundle exploration Coronoid fractures	Good view of the anterolateral facet of coronoid	Risk of neurovascular injury
Posterior (Olecranon osteotomy)	Distal humerus intra-articular fractures	Best exposure to the distal humerus	Non-union of the osteotomy site Need for hardware removal
Triceps reflecting/sparing approach	Distal humerus fractures Total elbow arthroplasty	Avoids olecranon osteotomy	Limited exposure Chance of triceps weakening, rupture
Posterior Boyd’s Approach	Access to radial head and neck (Alternative to Kocher, Kaplan, EDC split approach)	Can be extended distally with a lesser risk of PIN injury	PIN injury still may occur

A few of the common ones used include the Kocher, Kaplan, or extensor digitorum splitting intervals [10]. An abundance of literature support exists for lateral approaches to the radial head with predictable outcomes and complication rates [9]. The Kaplan interval lies between the ECRB and EDC whereas the Kocher interval lies more posteriorly between the ECU and anconeus. Extension of the lateral approaches is associated with injury to lateral ligamentous structures and excessive retraction of muscles around the radial neck particularly increases the risk of PIN injury [11]. Barnes et al. performed cadaveric dissections of the Kocher and Kaplan intervals and found the Kaplan interval to provide a significantly more surface area of access to the proximal radius than the Kocher interval [4]. In their short-term case series, Han et al. found the EDC splitting approach to the radial head to provide satisfactory functional results and noted no complications [12]. A recent cadaveric study showed that the EDC splitting approach provided better visualization of the anterior and anteromedial quadrant of the radial head as compared to the modified Kocher approach but similar visualization of the posterolateral quadrant [13].

As isolated radial head fractures more commonly involve the anterior or posterolateral quadrants and lateral approaches are commonly utilized due to the direct access to the radial head and predictable outcomes and complications [9,14]. When associated with coronoid fractures in terrible triad injuries, coronoid can be accessed by retracting the fractured radial head or a separate medial incision [9]. Problems arise when the radial head is associated with multiple injuries around the elbow. Greater soft tissue damage, more dissection, and a longer period of immobilization with multiple incisions are associated with a significantly higher rate of elbow stiffness [15]. These concerns are alleviated by utilizing a more extensive posterior approach to the elbow and particularly the radial head [16]. This single posterior approach may be utilized in cases that require two or more approaches to access the various injuries around the elbow.

Bryan and Morrey initially described an extensive posterior approach to the elbow joint with access to the olecranon, proximal radius, distal humerus, and ligamentous structures [17]. When the radial head fractures are associated with a Monteggia lesion, the proximal end of the olecranon may be opened in a shotgun maneuver (hyperflexion of the elbow) to deliver the radial head posteriorly [18]. However, when dealing with radial head fractures in isolation or terrible triad injuries, an interval is required to expose the radial head. Robinson et al. utilized a modification of the Boyd interval (fascia between anconeus and ECU incised followed by supinator osteotomy and release of the annular ligament, lateral collateral ligament (LCL), and capsule from the ulna) on 21 patients with radial head/neck fracture and associated injuries [10]. All patients had satisfactory flexion-extension and pronation-supination arc post-operatively with six cases of heterotopic ossifications during follow-up. The posterior approach provides good exposure to the lateral elbow structures with minimal risk of damage to the PIN [10]. A similar modified Boyd’s interval has been used in our series as well to approach fractures of the radial head. Although the PIN, which goes deep to the supinator, is not found during dissection, excessive supinator retraction must be avoided. A group comprising Landrum and colleagues published early results of the use of the anconeus approach (extensile posterior) in patients requiring radial head excision/replacement [16]. They retrospectively analyzed the results of a large cohort of 42 patients showing a satisfactory arc of motion at 40 weeks post-op with eight cases of heterotopic ossifications, one infection, and two patients requiring reoperation.

In our retrospective observational study, all subjects were treated for radial head fractures and associated injuries using the posterior approach. Although we encountered only one case of post-traumatic stiffness and ulnar nerve neuropraxia in our limited series, the complications of elbow surgery such as loss of motion, heterotopic ossifications, and post-traumatic arthritis must be kept in mind with the posterior approach as with other routine approaches. As the posterior capsule and lateral ligaments are released during exposure, there may also be a potential for elbow instability with this approach, but the prompt repair of these structures along with better access to coronoid and its repair leads to good post-operative stability.

The limitation of this study is the low sample size, lack of pre-operative scores, and the lack of a control group. Despite the incidence of complex elbow injuries being quite low, when occurred it necessitates greater exposure to managing them operatively. We have, with our study, shown good functional and radiological outcomes with the posterior approach. However larger studies with comparative analysis with other approaches as well as multi-centric studies with a larger population are required to further validate this technique and determine the incidence of PIN neuropraxia among the various approaches described.

## Conclusions

Radial head fracture is often associated with olecranon fracture or coronoid fracture with posterior dislocation. Instead of making a separate incision, we suggest that along with the radial head, olecranon fracture, coronoid fracture, and associated ligamentous injury can be addressed by a single posterior approach (Boyd) thereby decreasing soft tissue damage, early wound healing, and lesser chance of PIN damage.
